# Effective study selection using text mining or a single-screening approach: a study protocol

**DOI:** 10.1186/s13643-018-0839-x

**Published:** 2018-10-20

**Authors:** Siw Waffenschmidt, Elke Hausner, Wiebke Sieben, Thomas Jaschinski, Marco Knelangen, Inga Overesch

**Affiliations:** Institute for Quality and Efficiency in Health Care, Cologne, Germany

**Keywords:** Systematic reviews, Citation screening, Semi-automation, Screening prioritization

## Abstract

**Background:**

Systematic information retrieval generally requires a two-step selection process for studies, which is conducted by two persons independently of one another (double-screening approach). To increase efficiency, two methods seem promising, which will be tested in the planned study: the use of text mining to prioritize search results as well as the involvement of only one person in the study selection process (single-screening approach). The aim of the present study is to examine the following questions related to the process of study selection: Can the use of the Rayyan or EPPI Reviewer tools to prioritize the results of study selection increase efficiency? How accurately does a single-screening approach identify relevant studies? Which advantages or disadvantages (e.g., shortened screening time or increase in the number of full texts ordered) does a single-screening versus a double-screening approach have?

**Methods:**

Our study is a prospective analysis of study selection processes based on benefit assessments of drug and non-drug interventions. It consists of two parts: firstly, the evaluation of a single-screening approach based on a sample size calculation (11 study selection processes, including 33 single screenings) and involving different screening tools and, secondly, the evaluation of the conventional double-screening approach based on five conventional study selection processes. In addition, the advantages and disadvantages of the single-screening versus the double-screening approach with regard to the outcomes “number of full texts ordered” and “time required for study selection” are analyzed. The previous work experience of the screeners is considered as a potential effect modifier.

**Discussion:**

No study comparing the features of prioritization tools is currently available. Our study can thus contribute to filling this evidence gap. This study is also the first to investigate a range of questions surrounding the screening process and to include an a priori sample size calculation, thus enabling statistical conclusions. In addition, the impact of missing studies on the conclusion of a benefit assessment is calculated.

**Systematic review registration:**

Not applicable

## Background

The systematic screening of literature is a key component in systematic reviews. Stringent requirements exist for the transparency of the study selection process and the reliability of the corresponding results. These requirements aim to avoid the non-detection of relevant evidence with a subsequent risk of bias endangering the validity of conclusions based on the available evidence [1, 2].

Systematic information retrieval generally requires a two-step selection process for studies, which is conducted by two persons independently of one another (double-screening approach) [3–7]. This is one of a few methods known that might reduce the chance of missing relevant studies and is usually applied when screening the results of the bibliographic search. The double-screening approach has the following advantages: firstly, it can be ensured that the study inclusion criteria are applied consistently, thus avoiding systematic errors, and secondly, random errors such as careless mistakes can be identified and corrected. However, the approach is resource intensive, which can be a problem, as systematic reviews generally need to be completed within a defined period with a limited budget [1, 2].

To increase efficiency, two methods seem promising, which will be tested in our study: firstly, the use of text mining to prioritize search results and, secondly, the involvement of only one person in the study selection process (single-screening approach). Both methods can be used concurrently or separately in the study selection process.

### Prioritization through text mining

Various international research groups have investigated how information retrieval and study selection can be supported by technical aids [8]. Text mining is already being widely used in the development of search strategies and also seems to be a useful tool for prioritizing search results [1, 8]. Two different text-mining methods exist for the screening tools available, both of which are applied in the title and abstract screening process: “one aims to prioritize the list of items for manual screening so that the studies at the top of the list are those that are most likely to be relevant; the second method uses the manually assigned include/exclude categories of studies in order to ‘learn’ to apply such categorizations automatically” [1]. In addition to more efficient processing, a reduction in the overall number of citations retrieved would also save resources [1]. However, specifying a cut-off at which the selection process is stopped can be challenging [2].

Over the last few years, Internet-based screening tools such as Abstrackr [9], Rayyan [10], Covidence [11], and Eppi Reviewer [12] have been developed and are widely used. Some represent part of a comprehensive system for conducting a systematic review and contain additional functions, for example, for data extraction and meta-analysis (Covidence, EPPI Reviewer). For our study, we only consider those screening tools offering prioritization options [13]. To prepare for the study, we tested well-known screening tools and documented their advantages and disadvantages [14]. Our internal pre-study analysis showed that, in our opinion, three such tools are suitable for use in daily practice (Table 1), while for various reasons others (e.g., SWIFT, Distiller) are not. Two tools (Abstrackr, Rayyan) have recently been tested in explorative validation studies [2, 9], but to the best of our knowledge no studies directly comparing different tools exist [8]. We excluded AbstrackR, as in our opinion its future is unclear, and ultimately chose two tools with a prioritization option (Rayyan and EPPI Reviewer).

**Table 1:** Tools for prioritizing the results of the study selection process

Name	Link	Advantages	Disadvantages
Abstrackr	http://abstrackr.cebm.brown.edu	Easy to use	Future unclear, as tool was only developed by one person and the long-term support is unclear.
Rayyan	http://rayyan.qcri.org	Easy to use	Future development of this free software needs to be monitored.
Eppi Reviewer	http://eppi.ioe.ac.uk/eppireviewer4	Flexible options possible	Interface is not self-explanatory—working steps have to be read up in the 140-page handbook.

Rayyan and EPPI Reviewer both use a machine-learning algorithm to prioritize the order in which references are presented for screening. The ranking of references continuously improves as screening progresses and more manual decisions are available from which the algorithm can learn.

In Rayyan, the reviewers have to choose the “rating” option and the system assigns up to five stars to each reference. In our experience, the system starts to rank the citations after the reviewer has made eligibility decisions for about 50 citations.

In EPPI Reviewer 5, citations are ranked in their order of relevance after choosing “start priority screening.” A minimum of five relevant and five irrelevant reviewer decisions are needed before the machine-learning system is activated.

### Single-screening approach for study selection

A two-step selection process, that is, study selection on the title and abstract level followed by screening of the remaining citations on the full-text level, is an international standard [6, 7]. In addition, well-established handbooks recommend that two persons should be involved in the study selection process independently of one another to accurately identify relevant studies [5–7]. However, little robust evidence is available to support this recommendation [5–7]. The case study by Edwards 2002 [15] is mostly cited to justify this recommendation. Doust 2005 is a further case study [16]. Due to the inconsistent results of these two studies, both authors recommend the continued use of the double-screening approach. A further case study by Shemilt 2016 investigated four different screening methods (including single screening and single screening with text mining) for one topic and concluded that “alternatives to the conventional ‘double screening’ approach, integrating text mining, warrant further consideration” [17].

There is thus a need to systematically investigate a comprehensive amount of evidence to answer the question as to whether a double-screening approach is required for the study selection process. Even if one screener is enough to identify all relevant studies, this approach could entail disadvantages; for instance, a single screener might require much more time for screening because considerably more full texts are ordered. The choice between one or two screeners may also depend on their previous experience. As little evidence is available on these questions, they are also considered in our analysis.

## Methods/design

The aim of the present study is to examine the following questions related to the process of study selection from the results of the bibliographic search:

- Question 1: Can the use of the Rayyan or EPPI Reviewer tools for prioritizing the results of study selection increase efficiency?
- Question 2: How accurately does a single-screening approach identify relevant studies?
- Question 3: Which advantages or disadvantages (e.g., shortened screening time or increase in the number of full texts ordered) does a single-screening versus a double-screening approach have?

Our study is a prospective analysis of study selection processes based on benefit assessments of drug and non-drug interventions performed by the German Institute for Quality and Efficiency in Health Care (IQWiG). It consists of two parts: evaluation of a single-screening approach involving different screening tools and presentation of comparator data for the conventional double-screening approach.

### Data based on study selection processes using a single-screening approach

For the bibliographic search, study selection is tested by means of the original searches presented in the IQWiG benefit assessments. There is no restriction with regard to the study type considered. If an IQWiG project involves more than one search (e.g., one search for studies on the screening chain and a second for studies on the diagnostic accuracy of a screening test), each is analyzed separately.

**Fig. 1:**
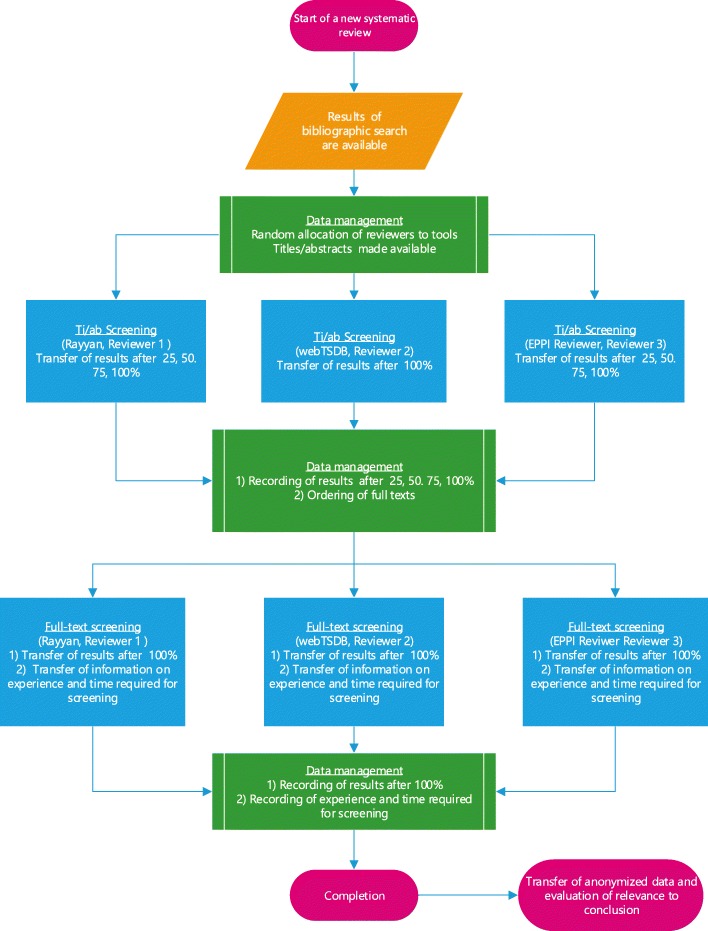
Process of study selection to evaluate the single-screening approach and the prioritization tools

Figure 1 illustrates the process of study selection. Each screener’s previous experience is recorded by means of the number of previous screenings or projects. In addition, each screener documents the time required for study selection. All projects and the corresponding searches involve three screeners, each allocated to IQWiG’s internal database webTSDB, the EPPI Reviewer, or Rayyan (see Table 2 for an example). Each screener screens all citations; screeners in EPPI Reviewer and Rayyan apply the prioritization function. On the basis of a sample size calculation, all searches in IQWiG projects involving a study selection process at the start of the project are included consecutively until a sample size of 11 is reached, so that 33 selection processes involving a single screener are considered in the analysis (see the “Information synthesis and analysis” section).

**Table 2:** Example of the allocation of screeners and tools to the searches under evaluation

Search no.	Screener no. 1	Screener no. 2	Screener no. 3
1	EPPI	webTSDB	Rayyan
2	webTSDB	EPPI	Rayyan
3	Rayyan	EPPI	webTSDB
4	EPPI	Rayyan	webTSDB
5	webTSDB	Rayyan	EPPI
6	Rayyan	webTSDB	EPPI etc.
…	…	…	

Before the selection process starts, each screener is given the project protocol with the study inclusion and exclusion criteria, as well as potentially relevant study publications and systematic reviews on the topic of interest, if available.

Screeners 1, 2, and 3 then independently screen all citations on the title and abstract level using the different tools. No consensus procedure takes places for studies with inconsistent classifications. All full texts included by at least one screener are ordered. The full texts are screened using the tools previously applied. The screener receives only those texts that he or she has classified as potentially relevant. Again, no consensus procedure is performed for studies with inconsistent classifications. The results of the selection process are recorded and evaluated separately for each screener.

On completion of the selection process, studies classified as relevant by at least one screener are allocated to the study pool and forwarded to the project group for further assessment. Further information sources used in addition to bibliographic databases are screened in the conventional way (e.g., search in study registries, scanning of reference lists, queries to manufacturers), and if applicable, additional citations are added to the study pool. The different project groups then further process the study pool (e.g., extraction of study characteristics, assessment of risk of bias). In this project phase, it may be decided for various reasons that certain studies are not eligible for further assessment (e.g., due to a lack of relevant outcome data) and are removed from the study pool retrospectively. The reference standard comprises only the relevant studies and publications identified in the bibliographic search and included in the final study pool. As stated, the final study pool may also include additional relevant studies identified by the search in further information sources. These studies are not included in the reference standard, but are included in the potential evaluation of the relevance of studies missed by single screeners for the conclusion of the benefit assessment (see section “outcomes,” questions 2 and 3). Data collection and analysis are anonymized and blinded, i.e., it is not disclosed which screener yielded which study pool with which tool.

### Data based on five additional conventional study selection processes using a double-screening approach

To obtain comparator data on the advantages and disadvantages with regard to the time required for screening and the number of full texts to be ordered, five additional conventional screening processes for the results of the bibliographic search are analyzed (a two-step and double-screening approach, with a consensus procedure for inconsistent citations after each step).

### Outcomes

The following outcomes are analyzed retrospectively to investigate the question of a potential increase in efficiency by using prioritization tools (question 1):

- Stop after screening 25%, 50%, or 75% of the publications (thresholds based on Olofsson 2017 [2]).
- Number of studies and publications included per prioritization tool and search that were identified despite a STOP criterion, related to the reference standard.
- Number of publications not needed to be screened with a STOP criterion.
- Calculate sensitivity (number of correctly identified relevant studies divided by the total number of relevant studies in the study pool) and specificity (number of correctly identified irrelevant studies divided by the total number of irrelevant studies).

The following outcomes are analyzed to investigate the question as to what extent each single screener identifies relevant studies (question 2):

- Number of relevant studies and publications identified. “Relevant” means either that all studies of the reference standard are identified, or that the studies not identified are not relevant to the conclusion of the benefit assessment.
- Calculate sensitivity (number of correctly identified relevant studies divided by the total number of relevant studies in the study pool).
- To evaluate this relevance, potential changes in the available evidence (i.e., changes in the study pool) and a subsequent potential change to the conclusion on the proof of benefit in the benefit assessment report are assessed for each outcome. If no such change is found for any outcome, the studies not identified in the selection process are classified as not relevant to the conclusion.

For all 16 screenings (11 with one and five with two screeners), the following outcomes are analyzed with regard to the advantages and disadvantages of the single-screening versus the double-screening approach (question 3):

- Number of full texts ordered
- Time required for study selection

### Information synthesis and analysis

The data on all questions are analyzed and presented using descriptive statistics.

The following specifications apply to the 33 single-screening processes (questions 1 and 2):

As each search involves three screeners and individual screeners can screen more than once, data dependencies exist. Sample size planning for this project roughly follows the confidence intervals (CIs) that can be reached (assuming data independency, which is not fulfilled) for the analyses described below.

For question 2, it is determined for each selection process whether all relevant studies are found and the following sample size calculations are conducted:

The probability that the study pool of a single screener includes all relevant studies is estimated by means of the relative frequency of selection processes yielding all relevant studies in relation to all selection processes. A one-sided CI according to Wilson is calculated for this proportion. If its lower limit is more than 90%, it is assumed that study selection by a single screener will yield a study pool of all relevant studies with sufficient certainty.

The planned number of 33 selection processes will allow a lower CI limit of 92.4% to be reached if all 33 processes contain all relevant studies (i.e., if no screener makes a mistake). If one mistake is made in 33 processes, then the 90% CI would be missed, as the lower CI limit would be 87.5%.

The following specifications apply to double-screening selection processes (question 3):

The outcomes investigated are analyzed in a purely descriptive manner and compared with the results of the 33 single-screening processes.

The previous work experience of the screener is considered as a potential effect modifier. If further potential effect modifiers are identified during the analysis, they can also be taken into account, as long as an explanation is provided.

## Discussion

### Challenges in study design

Simplified assumptions are made in order to enable the practical implementation of the study. For instance, various potential dependencies are not further considered. As stated, dependencies between the 33 single-screening processes may exist, as the same screener may be involved in several screening processes. Moreover, it is not taken into account whether the differences in the design of the various tools to be applied have an impact on the complete or incomplete identification of the study pool. In addition, instead of comparing a conventional double-screening approach with a single-screening approach, we summarize the screening results of the three single screeners as a reference standard. Considering such a comparison would require a substantial increase in resources (e.g. greater sample size, more screeners), a practical implementation of the study would be impossible. It should also be noted that we only consider previous screening experience, not clinical expertise, as a potential modifier for screeners. This is because at IQWiG, the researchers involved in screening generally have methodological expertise, but only rarely have clinical expertise. Clinical expertise is generally provided by external experts. In other organizations, screeners may also have clinical expertise, and this could represent a potential effect modifier.

Our simplified assumptions will be presented as a limitation of the study in the discussion section of the study publication.

### Strengths of the study design

Like in our study, the available evidence on prioritization using text mining is explorative. However, to the best of our knowledge, no study comparing the features of prioritization tools is currently available [1]. Our study can thus contribute to filling this evidence gap. Furthermore, other researchers will hopefully benefit from our work, as we will describe a practical way of using screening tools; this type of information is scarce.

The available evidence on single-screening processes for study selection is based on case studies. To the best of our knowledge, our study is the first to investigate a range of questions surrounding the screening process and also the first to include an a priori sample size calculation, thus enabling statistical conclusions. In addition, the impact of missing studies on the conclusion of a benefit assessment is calculated.

### Challenges in interpreting the results

Our study scrutinizes current methodological standards applied in systematic reviews: firstly, whether a double-screening selection process for studies is required, and secondly, whether by means of prioritization, the screening of all citations retrieved can be dispensed with without jeopardizing the completeness of the study pool. Our objective is to test methods (prioritization, single-screening selection process) that enable both an accurate and efficient study selection process. Their implementation would mean an increase in uncertainty, but possibly to a negligible extent. As Shemilt 2016 [17] concluded, such a decision depends on “the willingness of review teams and funders to sacrifice recall in order to substantively reduce the overall workload and total costs of systematic review production”. Besides the most important question, namely, whether all relevant studies are identified, further factors must be considered. These include the time required for screening, the number of full texts ordered, and the previous work experience of the screeners.

The particular challenge for our study is thus to provide a recommendation for a transparent and pragmatic method for the study selection process, despite the uncertainties to be expected.

## Abbreviations

CI: Confidence interval
IQWiG: Institute for Quality and Efficiency in Health Care
